# Guillain–Barre syndrome following scrub typhus: a case report and literature review

**DOI:** 10.1186/s12883-024-03645-9

**Published:** 2024-04-25

**Authors:** Shijun Hu, Zhichuan lin, Tao Liu, Shixiong Huang, Hui Liang

**Affiliations:** Department of Neurology, Hainan General Hospital, Hainan Affiliated Hospital of Hainan Medical University, Haikou, P. R. China

**Keywords:** Guillain–Barré syndrome, Anti-sulfatide antibodies, Scrub typhus, Case report, Neuropathy

## Abstract

**Background:**

Scrub typhus is an acute infectious disease caused by Orientia tsutsugamushi. Guillain–Barre syndrome (GBS) is an autoimmune-mediated peripheral neuropathy with a frequent history of prodromal infections, but GBS associated with scrub typhus is very rare.

**Case presentation:**

We report a 51-year-old male patient who developed dysarthria and peripheral facial paralysis following the cure of scfrub typhus. CSF examination and electrophysiological findings suggested a diagnosis of GBS. After treatment with intravenous immunoglobulin, the patient’s neurological condition improved rapidly.

**Conclusions:**

Scrub typhus infection is likely to be a potential predisposing factor in GBS, while scrub typhus-associated GBS has a favorable prognosis.

## Background

The etiological agent of scrub typhus is Orientia tsutsugamushi, which is characterized clinically by fever, eschar, splenomegaly, hepatomegaly, pneumonia, meningitis, and even multiple organ dysfunction [1]. Guillain–Barre syndrome (GBS) is an immune-mediated acute polyradiculoneuropathy associated with prodromal infections, injectable vaccines, malignancies, etc [2]. GBS has prodromal symptoms of infection that are mostly respiratory and gastrointestinal and is very rare in association with scrub typhus. To our knowledge, this is the first case of a GBS patient with elevated anti-sulfatide antibodies associated with scrub typhus infection.

## Case presentation

The patient was a 51-year-old male who was hospitalized due to fever for 10 days. The highest body temperature was approximately 39.0 ℃, and the fever had no obvious regularity, accompanied by head pain when fever was present. On examination, it was noted that there was an eschar over the right inguinal area, and several yellow bean-sized lymph nodes were palpable over the right inguinal area, with mild tenderness. Routine blood tests suggested a white blood cell count of 12.52 *10^9^/L, with 0% eosinophils. Meanwhile, metagenomic next-generation sequencing (mNGS) technology was used to detect pathogenic microbial DNA in blood, and as a result, 11,260 Orientia tsutsugamushi-specific sequences were detected. The patient was given intravenous treatment with doxycycline, and the patient was discharged after the resolution of symptoms of infection and continued oral doxycycline treatment as directed after discharge.

On the third day after discharge, also on the 15th day of presentation with febrile symptoms, the patient developed dysarthria, accompanied by salivation, weakness with closed eyes, and no limb weakness or numbness. Examination revealed that the patient had bilateral peripheral facial palsy, unclear articulation, dysphagia, pharyngeal reflexes were present, tendon reflexes of both lower limbs were reduced, and muscle strength of the four limbs was normal. The patient’s vital signs were uneventful, afebrile, conscious, bilateral pupils of consistent size, sensitive to light reflexes, and negative for signs of meningeal stimulation. Sensory and cerebellar examination was normal. CSF protein quantification was 2.04 g/L, white blood cell count was 6 *10^6^/L, CSF pressure, sugar and chloride were within normal ranges, and no pathogenic sequences were detected by mNGS of CSF. Serum anti-sulfatide IgM and IgG antibodies (western blot) were positive. CSF anti-sulfatide antibody IgG (western blot) was positive. Nerve conduction studies showed slowing of NCV in the peripheral nerves of all four limbs, mild prolongation of motor nerve conduction latencies, low distal SNAP amplitude, and marked prolongation of the F-wave latency in both median and tibial nerves in electrophysiological findings, suggesting demyelination. The patient was given intravenous administration of immunoglobulin (IVIG) therapy (0.4 g/kg/day) for 5 days, and after treatment, the patient’s neurological deficit improved significantly, and no significant sequelae were left at the follow-up visits.

## Discussion and conclusions

The patient presented with clinical manifestations of cranial neuropathy and albuminocytological dissociation in CSF with elevated anti-sulfatide antibodies in serum and CSF, which may be considered atypical GBS. Although the typical clinical picture of GBS syndrome with limb weakness and numbness was not present, decreased tendon reflexes in the lower extremities and peripheral neuropathy of the extremities suggested by electromyography were present.

Anti-sulfatide antibodies are associated with autoimmune neuropathy diseases and may play a role in demyelinating pathogenesis [3]. Elevated anti-sulfatide antibodies have been reported in cases of GBS [4]. Most of the patients with anti-sulfide antibody positivity had sensory or sensorimotor neuropathy in the distal extremities, and only a few of them had demyelinating neuropathy [5]. Although this patient with positive anti-sulfide antibodies had cranial nerve damage as the primary clinical manifestation, a nerve conduction study was suggestive of the presence of a demyelinating peripheral neuropathy involving motor and sensory nerves in the extremities. In fact, patients with anti-sulfatide IgM antibody positivity often present different neurophysiological and pathological findings and therapeutic responses in the peripheral nervous system [6].

Scrub typhus, a febrile disease caused by the gram-negative coccal bacterium Orientia tsutsugamushi, is transmitted to humans through the bites of infected larval mites and is commonly characterized clinically by fever, eschar, rash, splenomegaly, hepatomegaly, and lymphadenopathy and, in severe cases, meningoencephalitis, multiorgan dysfunction, and disseminated intravascular coagulation [1, 7]. Scrub typhus infection can usually involve the central nervous system, and approximately one-fifth to three-thirds of patients with scrub typhus suffer from neurological complications, but the appearance of symptoms of peripheral neuropathy is very rare [8–10]. A summary of sixteen case reports of GBS associated with scrub typhus, including the present case, is provided in Table 1 [11–20]. Among all the cases with available details of medical history, all patients developed GBS-related symptoms at least 7 days after presenting symptoms of scrub typhus infection and most concentrated at 10 to 17 days, with a mean of 10.6 days. The mean age of the reported patients was 46 years (7–74 years), and most of them were men (12 men out of 16 cases: 75%). Symptoms of these scrub typhus-associated cases of GBS include muscle weakness, cranial nerve palsy, hypotonia, decreased or absent tendon reflexes, sensory disturbances, abducens nerve palsy, and in severe cases, respiratory failure and disturbance of consciousness. For patients with GBS and persistent scrub typhus infection, fever and meningeal irritation signs may be positive. Albuminocytological dissociation (by the Brighton criteria [21]) was present in 14 of these 16 patients with CSF results and in 2 with CSF results within the normal range, although it is not clear on the basis of the current data that these CSFs were sampled in the weeks after the onset of peripheral neurological symptoms.

**Table 1:** Summary of case reports of Guillain Barre syndrome associated with scrub typhus

Case Author (year)	Age (yrs)/Sex	Onset^a^ (day-weeks)	Neuropathic symptoms	Findings	Nerve conduction study	Treatments	Outcomes
1 Lee et al. [11] (2007)	42/F	2 weeks	quadriparesis, facial diplegia, areflexia	albuminocytological dissociation in CSF^b^	demyelinating neuropathy^c^	IVIg	improvement
2 Lee et al. [12] (2009)	54/m	17 days	facial paralysis, quadriparesis, hyporeflexia.	CSF normal	prolonged F-responses, slowed NCV	doxycycline, prednisolone, IVIg	improvement
3 Lee et al. [12] (2009)	74/F	10 days	quadriparesis, hyporeflexia.	CSF normal.	prolonged distal latencies, slowed NCV and reduced amplitude of CMAP	antibiotics, prednisolone, IVIg	improvement
4 Ju et al. [13] (2011)	46/F	7 days	quadriparesis, disturbance of consciousness, respiratory failure, hyporeflexia	albuminocytological dissociation in CSF^b^	acute sensoriomotor polyneuropathy^c^	antibiotics, mechanical ventilation, rehabilitation therapy	improvement
5 Ju et al. [13] (2011)	60/m	8 days	quadriparesis, disturbance of consciousness, dysphagia, sensory disturbance, respiratory failure, hyporeflexia	GM1 IgM and GD1b IgM antibodies positive, albuminocytological dissociation in CSF^b^	diffuse demyelinated neuropathy^c^	IVIg, doxycycline, mechanical ventilation	improvement
6 Sawale et al. [14] (2014)	41/m	15 days	quadriparesis, facial paresis, hypotonia, the anterior abdominal muscles and paraspinal muscles weakness; deep tendon areflexia	albuminocytological dissociation in CSF^b^	absent F-waves, prolonged latency, slowed NCV and reduced amplitude of CMAP	plasmapheresis	improvement
7 Kim et al. [15] (2014)	70/m	2 weeks	ophthalmoplegia with bilateral ptosis, facial diplegia, gait ataxia, areflexia	albuminocytological dissociation in CSF^b^	reduced SNAP and absent H-reflexes	IVIg	improvement
8 Sakai et al. [19] (2016)	66/m	7 days	quadriparesis, dysphagia, sensory disturbance, hyporeflexia	albuminocytological dissociation in CSF^b^, IgM-GD1a、IgM-GT1b、IgM-GalNAc-GD1a Positive	prolonged distal latencies, sensory potential not drawn out	IVIg	improvement
9 Sakai et al. [19] (2016)	58/F	15 days	quadriparesis, facial diplegia, sensory disturbance, hyporeflexia, respiratory failure	albuminocytological dissociation in CSF^b^	prolonged distal latencies, reduced amplitude of CMAP, sensory potential not drawn out	IVIg, mechanical ventilation	improvement
10 Gangula et al. [16] (2017)	40/m	10 days	hypotonia, areflexia quadriparesis	plasmodium falciparum coinfection; albuminocytological dissociation in CSF^b^	reduced amplitude of SNAP、 CMAP, absent F-waves, prolonged distal latency, slowed NCV	antimalarials, antibiotics, physiotherapy	improvement
11 Dev et al. [17] (2019	20/m	8 days	quadriparesis, hypotonia, deep tendon areflexia	leptospirosis coinfection, albuminocytological dissociation in CSF^b^	acute inflammatory demyelinating polyradiculo-neuropathy^c^	antibiotics, supportive measures	improvement
12 Juneja et al. [18] (2020)	42/m	7 days	quadriparesis, hypotonia, trunk and neck muscles weakness, deep tendon areflexia	albuminocytological dissociation in CSF^b^	prolonged distal latencies, reduced NCV, and absent F responses	IVIg	improvement
13 Pandey et al. [20] (2021)	56/m	1 week	quadriparesis, facial diplegia, abducens nerve palsy, areflexia	albuminocytological dissociation in CSF^b^	demyelinating patterns of motor neuropathies^c^	doxycycline, IVIg, supportive measures	improvement
14 Raghunathan et al. [24](2022)	7/m	1 week	quadriparesis, areflexia, truncal weakness disturbance of consciousness, neck stiffness	albuminocytological dissociation in CSF^b^	absent H-reflexes, absent F responses	IVIg, azithromycin	improvement
15 Modi et al. [25] (2023)	20/m	10 days	fever, disturbance of consciousness, quadriparesis, deep tendon areflexia, respiratory failure	albuminocytological dissociation in CSF^b^.	reduced amplitude of CMAP	doxycycline, IVIg, mechanical ventilation, rehabilitation therapy	improvement
16 The present case	51/m	15 days	facial diplegia, unclear articulation, dysphagia, hyporeflexia	albuminocytological dissociation in CSF^b^. anti-sulfatide antibodies positive.	slowed NCV, prolonged CMAP, reduced amplitude of SNAP, prolonged F- waves	IVIg	improvement

^a^ Temporal onset of time from fever or other symptoms associated with scrub typhus to the appearance of symptoms of peripheral nerve impairment

^b^ Albuminocytological dissociation in CSF: elevation of cerebrospinal protein above the laboratory normal AND total CSF white cell count <50 cells/µl according to the Brighton criteria [21]

^c^ No detailed examination results available

Abbreviations: intravenous immunoglobulin: IVIg; SNAP: sensory nerve action potential; CMAP: compound muscle action potential; NCV: nerve conduction velocity

From Table 1, 10 patients developed GBS-related symptoms at the same time as scrub typhus infection, and the remaining 6 patients developed symptoms after the cure of scrub typhus. Among all the cases with available treatment details, 10 patients had concomitant scrub typhus infection, 3 used antibiotics, 2 used antibiotics, IVIg and prednisolone, 4 used antibiotics and IVIg, and 1 used IVIg (unclear whether antibiotics were used). Of the 6 patients with GBS following scrub typhus infection, 5 received IVIg, and 1 received plasmapheresis. Four patients used mechanical ventilation because of respiratory failure due to respiratory muscle weakness. This suggests that antibiotic therapy is essential in cases of GBS with persistent scrub typhus infection, that the addition of prednisolone and immunoglobulin may be considered when treatment is suboptimal and that treatment with immunoglobulins or plasmapheresis can be routinely administered in cases of GBS following scrub typhus infection. All the reviewed patients experienced near complete improvement of their peripheral nerve symptoms, and none died or had sequelae affecting daily tasks, implying a better prognosis for scrub typhus-associated GBS.

Numerous infectious agents have been associated with the onset of GBS and consequently with the production of antiganglioside antibodies [22]. Antiganglioside antibodies are detected in more than half of GBS patients, and their detection strongly supports the diagnosis of GBS [23]. Although antiganglioside antibodies assist in GBS diagnosis, their signal is not strong in GBS caused by scrub typhus infection. Only two patients out of 7 cases in which ganglioside-related antibody testing was performed were positive [11, 13, 15, 19, 20]. Furthermore, only GM1, GD1a, GD1b, GT1b, GalNAc-GD1a and anti-sulfatide antibodies were detected in the current reviewed cases of GBS associated with scrub typhus infection. The underlying mechanism may be an immune-mediated type 2 hypersensitivity reaction against self-antigens [10].

In conclusion, scrub typhus infection is likely to be a potential predisposing factor in GBS, although the immunological mechanisms involved are not clear. The possibility of GBS should be considered in patients who present with symptoms of peripheral neuropathy at the time of scrub typhus infection or after a recent cure. Meanwhile, assessment of whether scrub typhus infection is still present may be necessary. The timely use of antibiotics to treat scrub typhus may improve the symptoms of GBS. For GBS after the cure of tsutsugamushi disease, treatment means such as IVIg or plasmapheresis receive relatively good results. Finally, combined with the current literature that has been reviewed, scrub typhus-associated GBS has a favorable prognosis.

## Data Availability

Not applicable.
